# Qualitative analysis and chaotic behavior of respiratory syncytial virus infection in human with fractional operator

**DOI:** 10.1038/s41598-023-51121-0

**Published:** 2024-01-25

**Authors:** Saba Jamil, Abdul Bariq, Muhammad Farman, Kottakkaran Sooppy Nisar, Ali Akgül, Muhammad Umer Saleem

**Affiliations:** 1 Institute of Mathematics, Khwaja Fareed University of Engineering and Information Technology, Rahim Yar Khan, Pakistan; 2 Department of Mathematics, Laghman University, Mehtarlam, 2701 Laghman Afghanistan; 3 Department of Mathematics, Faculty of Arts and Sciences, Near East University, Mersin, Turkey; 4 Department of Computer Science and Mathematics, Lebanese American University, Beirut, Lebanon; 5 Department of Mathematics, College of Science and Humanities in Alkharj, Prince Sattam Bin Abdulaziz University, Alkharj, Saudi Arabia; 6 Department of Mathematics, Art and Science Faculty, Siirt University, 56100 Siirt, Turkey; 7 Department of Mathematics, University of Education, Lahore, Pakistan

**Keywords:** Biophysics, Mathematics and computing

## Abstract

Respiratory syncytial virus (RSV) is the cause of lung infection, nose, throat, and breathing issues in a population of constant humans with super-spreading infected dynamics transmission in society. This research emphasizes on examining a sustainable fractional derivative-based approach to the dynamics of this infectious disease. We proposed a fractional order to establish a set of fractional differential equations (FDEs) for the time-fractional order RSV model. The equilibrium analysis confirmed the existence and uniqueness of our proposed model solution. Both sensitivity and qualitative analysis were employed to study the fractional order. We explored the Ulam–Hyres stability of the model through functional analysis theory. To study the influence of the fractional operator and illustrate the societal implications of RSV, we employed a two-step Lagrange polynomial represented in the generalized form of the Power–Law kernel. Also, the fractional order RSV model is demonstrated with chaotic behaviors which shows the trajectory path in a stable region of the compartments. Such a study will aid in the understanding of RSV behavior and the development of prevention strategies for those who are affected. Our numerical simulations show that fractional order dynamic modeling is an excellent and suitable mathematical modeling technique for creating and researching infectious disease models.

## Introduction

A common respiratory virus is the single-stranded RNA virus known as respiratory syncytial virus (RSV). RSV is a cause of illnesses of the respiratory system, including infections of the middle ear, lungs, and airways. The common cold, bronchitis, croup, etc. are all most frequently brought on by it. Certain people are typically severely infected by this virus, particularly pretermit infants, elderly people, infants, adults with heart and lung conditions, and someone with a highly weakened defense mechanism^1,2^. The infection may transferred through actual interaction with contagious excretions or through droplets released during a person’s cough. It can be spread unintentionally by contact with hands that have touched dining utensils or any items that have been recently soiled by a sick person’s nasal or throat discharge. In other words, the infection spreads quickly. In the United States, about $60\%$ of newborns contract HRSV during their first season, and the virus will largely attack children between the ages of two and three^3^.

Mathematical models can be used to predict the emergence of infectious diseases, as is well known. Finding the epidemic’s anticipated outcome in this way is helpful for the objectives of public health initiatives. As a fundamental mathematical framework, compartmental models can be used to investigate the intricate dynamics of epidemiological systems^4^. Recently, several important efforts have been made to execute this inquiry program for several disorders with integer-order compartmental models, including cholera^5^, hepatitis B^6^, zika virus^7^, malaria transmission^8^, dengue^9^, and influenza^10^. A detailed model of HRSV transmission was presented in^2^, in which individuals gradually develop immunity to the infection after being exposed to it repeatedly. In this study, a comparison between the sophisticated model indicated above and a typical SIRS framework was also made. In^11^, the HRSV was modeled using an age-structured mathematical formalism that specifically took into account the youngest patients, or children under the age of one, who are most impacted by the disease. A numerical method for solving the HRSV seasonal model was presented in^12^. Sungchasit et al.^13^ described the global stability (GS) analysis of the super spreading RSV disease.

In the literature, fractional differential equations (FDEs), also known as significant DEs, are an extension of differential equations that use fractional calculus, a branch of mathematical analysis that looks at alternative approaches to creating differentiation operators of non-integer order^14,15^. Due to their natural connection to memory-based systems, FDEs are advantageous in the majority of biological systems^16–18^. Through fractional derivatives, Jan et al.^19^ build an epidemic model for Rift Valley fever with vaccination. In the paper^20^, the authors examined a dengue infection model with partial immunity and asymptomatic subjects. A compartmental model for the dengue fever transmission phenomenon was presented by Jan et al.^21,22^ and included nonlinear forces of infection via a fractional derivative. In^23^, scientists examined tumor-immune interactions using a fractional derivative framework. A unique mathematical model for the transmission of HFMD with reinfection was developed by researchers in^24^. In reference^25^, researchers developed a mathematical model using the Atangana–Baleanu operator within fractal fractional-order principles to depict the transmission of pneumonia among a population. Several epidemiological models have included FDEs. For more applications see^26–30^.

In many different systems and processes, FDs are an effective tool for explaining memory and hereditary characteristics. The fundamental details of the function are preserved in stacked form via fractional-order differential equations. In fractional-order modeling, the order of the derivative is one such additional variable we have that is helpful for numerical techniques. The dynamics of disease transmission have been investigated using fractional-order modeling. Additionally, the fractional differentiation is not local, while the integer-order differentiation is. The simulation of epidemic scenarios is aided by this behavior. Additionally, the fractional derivative can expand the system’s stability zone. Due to its ability to incorporate common starting and boundary conditions into the derivation and the fact that the derivative of a constant is zero, as opposed to the fractional Riemann–Liouville derivative, the Caputo derivative is highly useful when discussing real-world concerns. The aforementioned investigations as well as the aid of Caputo FDEs served as inspiration for this study. A fractional order $SEI_rI_sR$ model was developed utilizing Caputo FDEs to characterize the dynamics of RSV.

These are the goals of this work:

- Examine the stability and dynamical behavior of the $SEI_rI_sR$ model.
- The Basic Reproduction number and Equilibrium points should be determined.
- Application of the Lagrange polynomial technique to obtain a numerical solution.
- The fractional order RSV model is demonstrated with chaotic behaviors.

This paper is structured as follows: section one serves as an introduction, while section two presents fundamental fractional order derivatives applicable to solving the epidemic model. The third section delves into the positivity of the fractional order model, discussing endemic equilibrium, DFE, and sensitivity analysis. In “Qualitative analysis of model”, the existence and uniqueness of a system of model solutions are affirmed using the fixed point concept. “Ulam–Hyers stability” focuses on investigating the Ulam–Hyers stability of the RSV model. “Numerical scheme” explores the impact of fractional parameters by employing numerical techniques to solve the fractional-order system. Finally, “Simulation and discussion” and “Conclusion” discuss the results and conclusion.

## Basic concepts

Before creating the model, we must first study the fundamental definitions that are essential to comprehending fractional operators.

### Definition 2.1

^14^Assume that $w(t) \in {H^1}(0,T), \ T > 0,$ and $0 < \beta \le 1$. The following is the definition of a power-law kernel fractional derivative:

1 $$\begin{aligned} ^{C}D_{t}^{\beta {}}w(t) = \frac{1}{{\Gamma (1 - \beta )}}\frac{d}{{d{t{{}}}}}\int \limits _0^t {{{(t - \xi )}^{- \beta }}w(\xi )d\xi .} \end{aligned}$$

### Definition 2.2

^14^For (1), the corresponding fractional integral operator is given by

2 $$\begin{aligned} ^{C}I_{t}^{\beta {}}w(t) = \frac{1}{{\Gamma (\beta )}}\int \limits _0^t {{{(t - \xi )}^{\beta - 1}}{ { {}}}w(\xi )d\xi .} \end{aligned}$$

## Fractional order RSV model

Here, we propose an RSV model with memory-affected fractal fractional order. The RSV epidemic model presented in^13^ is a classical derivative that has to be taken into account. The entire population is classified into five classes: susceptible *S*(*t*), exposed *E*(*t*), normally infectious $I_r(t)$, super infectious $I_s(t)$, and recovered *R*(*t*). The birth rate is indicated by *b* while the natural death rate is symbolized by $\mu$. The overall size of the human population at a given time is denoted by *N*. The rate of virus transmission between people, which affects the disease’s spread is represented by the symbol $\beta$. The interval from infection to the development of symptoms, or the incubation time of the virus within an infected human, is shown by the symbol $\eta$. The likelihood that a new case of the virus will manifest as a normal infected human, typically characterized by typical transmission patterns indicated by *p*. The additional likelihood that a new case may involve an infected person who is super-spreading, who has an elevated potential for transmitting the virus to a larger number of individuals indicated by $(1-p)$. The rate at which individuals with normal infections recover from the disease is symbolized by $r_1$. The rate at which individuals with super-spreading infections recover is indicated by $r_2$. The set of non-linear fractional differential equations is

3 $$\begin{aligned} \begin{array}{l} ^CD_t^\alpha S(t) = bN - \beta S(t)I(t) - \mu S(t),\\ ^CD_t^\alpha E(t) = \beta S(t)I(t) - \left( {\frac{1}{\eta }} \right) pE(t) - \left( {\frac{1}{\eta }} \right) \left( {1 - p} \right) E(t) - \mu E(t),\\ ^CD_t^\alpha {I_r}(t) = \left( {\frac{1}{\eta }} \right) pE(t) - {r_1}{I_r}(t) - \mu {I_r}(t),\\ ^CD_t^\alpha {I_s}(t) = \left( {\frac{1}{\eta }} \right) \left( {1 - p} \right) E(t) - {r_2}{I_s}(t) - \mu {I_s}(t),\\ ^CD_t^\alpha R(t) = {r_1}{I_r}(t) + {r_2}{I_s}(t) - \mu R(t),\end{array} \end{aligned}$$

the initial conditions are

$$\begin{aligned} S(t) \ge 0,\;E(t) \ge 0,\;{I_r}(t) \ge 0,\;{I_s}(t) \ge 0,R(t) \ge 0. \end{aligned}$$

### Steady state analysis

Here, we will look into the RSV fractional-order system for both endemic and disease-free steady states. Setting the fractional derivative ${}^CD_t^\alpha S$, ${}^CD_t^\alpha E$, ${}^CD_t^\alpha {I_r}$, ${}^CD_t^\alpha {I_s}$ and ${}^CD_t^\alpha R$ of the fractional system (3) without infection to zero allows us to reach the steady-state with no infection. Disease free equilibrium points are

4 $$\begin{aligned} {D^0}=({S^0},\ {E^0}, \ {I_r^0}, \ {I_s^0}, \ {R^0}) = \left( \frac{{Nb}}{\mu },\ 0, \ 0,\ 0,\ 0\right) . \end{aligned}$$

The endemic equilibrium can be expressed as follows by setting the right-hand side of the system (3) to zero and assuming that none of the disease states is zero,

$$\begin{aligned} {D^*}=({S^*},\ {E^*}, \ {I_r^*}, \ {I_s^*}, \ {R^*}), \end{aligned}$$

where

$$\begin{aligned} {S^ * }= & {} \frac{{\left( {(\mu + {r_1})(\mu + {r_2})(\eta \mu + 1} \right) }}{{\left( {\beta (\mu + {r_1} - p{r_1} + p{r_2})} \right) }},\\ {E^ * }= & {} \frac{{\left( {\eta \left( {Nb\beta (\mu + {r_1} - p{r_1} + p{r_2}) - ({\mu ^3} + {r_1}{r_2}\mu )(1 + \eta \mu ) - ({\mu ^2} + \eta {\mu ^3})({r_1} + {r_2})} \right) } \right) }}{{\beta \left( {\eta \mu + 1} \right) \left( {\mu + {r_1} - p{r_1} + p{r_2}} \right) }},\\ {I_{r}^ * }= & {} \frac{{\left( {p\left( {Nb\beta (\mu + {r_1} - p{r_1} + p{r_2}) - ({\mu ^3} + {r_1}{r_2}\mu )(1 + \eta \mu ) - ({\mu ^2} + \eta {\mu ^3})({r_1} + {r_2})} \right) } \right) }}{{\beta \left( {\mu + {r_1}} \right) \left( {\eta \mu + 1} \right) \left( {\mu + {r_1} - p{r_1} + p{r_2}} \right) }},\\ {I_{s}^ * }= & {} \frac{{\left( {\left( {p - 1} \right) \left( {({\mu ^3} + {r_1}{r_2}\mu )(1 + \eta \mu ) + ({\mu ^2} + \eta {\mu ^3})({r_1} + {r_2}) - Nb\beta (\mu + {r_1} - p{r_1} + p{r_2})} \right) } \right) }}{{\beta \left( {\mu + {r_2}} \right) \left( {\eta \mu + 1} \right) \left( {\mu + {r_1} - p{r_1} + p{r_2}} \right) }},\\ {R^ * }= & {} \frac{{\left( {\left( {\mu {r_2} + {r_1}{r_2} + \mu p{r_1} - \mu p{r_2}} \right) \left( {Nb\beta (\mu + {r_1} - p{r_1} + p{r_2}) - ({\mu ^3} + {r_1}{r_2}\mu )(1 + \eta \mu ) - ({\mu ^2} + \eta {\mu ^3})({r_1} + {r_2})} \right) } \right) }}{{\beta \mu \left( {\mu + {r_1}} \right) \left( {\mu + {r_2}} \right) \left( {\eta \mu + 1} \right) \left( {\mu + {r_1} - p{r_1} + p{r_2}} \right) }}. \end{aligned}$$

Utilizing the next-generation technique, we were able to determine the system’s fundamental reproduction number, which is represented by $R_0$. In^13^ is given as:

5 $$\begin{aligned} {R_0} = \frac{{b\beta {\Psi _1}}}{{{\Psi _2}}}, \end{aligned}$$

where ${\Psi _1} = {r_1} + p{r_1} + p{r_2} + \mu + 2p\mu ,$ and ${\Psi _2} = \mu \left( {\mu + {r_1}} \right) \left( {\mu + {r_2}} \right) \left( {\eta \mu + 1} \right) .$ The aforementioned $R_0$ serves as a threshold parameter, if $R_0$ is less than 1, the disease disappears, and if $R_0$ is more than 1, the contagion perseveres in the community.

### Sensitivity analysis

We can check the sensitivity of $R_0$ by computing the partial derivative for the significant variables, that is:

$$\begin{aligned}{} & {} \frac{{\partial {R_0}}}{{\partial b}} = \frac{{\beta {\Psi _1}}}{{{\Psi _2}}}> 0,\\{} & {} \frac{{\partial {R_0}}}{{\partial \beta }} = \frac{{b{\Psi _1}}}{{{\Psi _2}}}> 0,\\{} & {} \frac{{\partial {R_0}}}{{\partial {r_1}}} = \frac{{ - b\beta p}}{{\left( {\mu {{\left( {\mu + {r_1}} \right) }^2}\left( {\eta \mu + 1} \right) } \right) }}< 0,\\{} & {} \frac{{\partial {R_0}}}{{\partial p}} = \frac{{\left( {b\beta \left( {2\mu + {r_1} + {r_2}} \right) } \right) }}{{{\Psi _2}}} > 0,\\{} & {} \frac{{\partial {R_0}}}{{\partial {r_2}}} = \frac{{ - b\beta \left( {p + 1} \right) }}{{\left( {\mu {{\left( {\mu + {r_2}} \right) }^2}\left( {\eta \mu + 1} \right) } \right) }}< 0,\\{} & {} \frac{{\partial {R_0}}}{{\partial \mu }} = \frac{{b\beta \left( {2p + 1} \right) }}{{{\Psi _2}}} - \frac{{b\beta {\Psi _1}}}{{\mu {{\left( {\mu + {r_1}} \right) }^2}\left( {\mu + {r_2}} \right) \left( {\eta \mu + 1} \right) }}\\{} & {} \quad - \frac{{b\beta {\Psi _1}}}{{{\mu ^2}\left( {\mu + {r_1}} \right) \left( {\mu + {r_2}} \right) \left( {\eta \mu + 1} \right) }} - \frac{{b\beta {\Psi _1}}}{{\mu \left( {\mu + {r_1}} \right) {{\left( {\mu + {r_2}} \right) }^2}\left( {\eta \mu + 1} \right) }}\\{} & {} \quad - \frac{{b\beta {\Psi _1}}}{{\mu \left( {\mu + {r_1}} \right) \left( {\mu + {r_2}} \right) {{\left( {\eta \mu + 1} \right) }^2}}}< 0,\\{} & {} \frac{{\partial {R_0}}}{{\partial \eta }} = \frac{{ - b\beta {\Psi _1}}}{{\left( {\mu + {r_1}} \right) \left( {\mu + {r_2}} \right) {{\left( {\eta \mu + 1} \right) }^2}}} < 0. \end{aligned}$$

$R_0$ is extremely responsive to changes in the parameters. In this work, the values *b*, $\beta$, and *p* are increasing, while $r_1$, $r_2$, $\mu$, and $\eta$ are decreasing. To avoid an illness, prevention is preferable to treatment.

#### Theorem 3.1

*The closed set* $\Phi = \left\{ {\left( {S, E,{I_r},{I_s}, R} \right) \in R_ + ^5:N \le \frac{b}{\mu }} \right\}$ *is a positive invariant set for the proposed fractional-order system* (3).

#### *Proof*

To prove that the system of Eqs. (3) has a non-negative solution, we have

6 $$\begin{aligned} \begin{array}{l} {\left. {^CD_t^\alpha S(t)} \right| _{S = 0}} = bN \ge 0,\\ {\left. {^CD_t^\alpha E(t)} \right| _{E = 0}} = \beta S(t)I(t) \ge 0,\\ {\left. {^CD_t^\alpha {I_r}(t)} \right| _{{I_r} = 0}} = \left( {\frac{1}{\eta }} \right) pE(t) \ge 0,\\ {\left. {^CD_t^\alpha {I_s}(t)} \right| _{{I_s} = 0}} = \left( {\frac{1}{\eta }} \right) \left( {1 - p} \right) E(t) \ge 0,\\ {\left. {^CD_t^\alpha R(t)} \right| _{R = 0}} = {r_1}{I_r}(t) + {r_2}{I_s}(t) \ge 0.\\ \end{array} \end{aligned}$$

Thus, the fractional system (3) has non-negative solutions. Lastly By adding all the relations of the system (3), the total population with the fractional derivative is given as

$$\begin{aligned} {}^CD_t^\alpha N(t)&= bN - \mu N\\&\le b - \mu N. \end{aligned}$$

Take a Laplace transform to both sides, we get

$$\begin{aligned} N(s) \le \frac{b}{{s({s^\alpha } + \mu )}} - N(0)\frac{{{s^{\alpha - 1}}}}{{{s^\alpha } + \mu }}. \end{aligned}$$

Take Laplace inverse to both sides and Theorem 7.2 in^31^, we obtained

7 $$\begin{aligned}{} & {} N(t) \le N(0){E_\alpha }\left( { - \mu {t^\alpha }} \right) + \int \limits _0^t {b{\sigma ^{\alpha - 1}}{E_{\alpha ,\alpha }}\left( { - \mu {\sigma ^\alpha }} \right) } d\sigma ,\nonumber \\{} & {} N(t) \le N(0){E_\alpha }\left( { - \mu {t^\alpha }} \right) + \int \limits _0^t {b{\sigma ^{\alpha - 1}}\sum \limits _{j = 0}^\infty {\frac{{{{\left( { - 1} \right) }^j}{\mu ^j}{\sigma ^{j\alpha }}}}{{\Gamma \left( {j\alpha + \alpha } \right) }}} } d\sigma \nonumber \\{} & {} \quad = \frac{b}{\mu } + {E_\alpha }\left( { - \mu {t^\alpha }} \right) \left( {N(0) - \frac{b}{\mu }} \right) . \end{aligned}$$

Because of this if $N(0) \le \frac{b}{\mu }$ then for $t > 0$, $N(t) \le \frac{b}{\mu }$. Therefore, in the context of fractional derivative, positive invariance exists for the closed set $\Phi$. $\square$

#### Remarks 3.1

The closed set $\Phi$ is currently representing a set of conditions that are biologically significant in the context of RSV transmission modeling. It is emphasizing the relationship between population dynamics, birth and death rates, and the vulnerability of specific age groups. These insights are currently informing strategies for managing and controlling RSV transmission within a population.

#### Theorem 3.2

*If* $R_0 < 1,$ *the DFE of the system* (3) *is LAS otherwise unstable*.

#### *Proof*

The system’s (3) Jacobian matrix at $D^0$

8 $$\begin{aligned}{} & {} J = \left[ {\begin{array}{ccccccccccccccc}{ - \mu }&{}0&{}{ - \beta \frac{{{S^0}}}{N}}&{}{ - \beta \frac{{{S^0}}}{N}}\\ 0&{}{ - \frac{1}{\eta }p - \left( {\frac{1}{\mu }} \right) \left( {1 - p} \right) - \mu }&{}{\beta \frac{{{S^0}}}{N}}&{}{\beta \frac{{{S^0}}}{N}}\\ 0&{}{\frac{1}{\eta }p}&{}{ - {r_1} - \mu }&{}0\\ 0&{}{\left( {\frac{1}{\mu }} \right) \left( {1 - p} \right) }&{}0&{}{ - {r_2} - \mu }\end{array}} \right] ,\nonumber \\{} & {} \left| {J\left( {{D^0}} \right) - \lambda I} \right| = 0,\nonumber \\{} & {} {\lambda _1} + \mu = 0, \end{aligned}$$

and

9 $$\begin{aligned} {\lambda ^3} + {d_1}{\lambda ^2} + {d_2}\lambda + {d_3} = 0, \end{aligned}$$

where

$$\begin{aligned} {d_1}= & {} \frac{1}{\eta } + {r_1} + {r_2} + 3{\mu ^2},\\ {d_2}= & {} \frac{1}{\eta }\left( { - \frac{{{S^0}}}{N}\beta + \left( {{r_1} + {r_2} + 2\mu + \left( {{r_1}{r_2} + 2\mu \{ \left( {{r_1} + {r_2}} \right) + 3{\mu ^2}} \right) \} \eta } \right) } \right) ,\\ {d_3}= & {} \frac{1}{\eta }\left( {\frac{{{S^0}}}{N}\beta \left( {\left( { - 1 + p} \right) {r_1} - p{r_2} - \mu } \right) + \left( {{r_1} + \mu } \right) \left( {{r_2} + \mu } \right) \left( {1 + \mu \eta } \right) } \right) . \end{aligned}$$

It should be emphasized that the suggested model assumes positive values for the parameters. Therefore, the eigenvalue $\lambda _1 < 0$. Indeed the quantity $\mu$ is strictly positive. Thus, there are no positive roots of Eq. (9) according to Descarte’s rule of signs because there is no sign change if $R_0<1$. In addition, if $\lambda$ is changed to $- \lambda$ in Eq. (9), then Eq. (9) has three signs that change if $R_0 < 1,$ therefore there are precisely three negative roots to Eq. (9). Thus by the condition $\left| {\arg {\lambda _i}} \right| > \frac{{\alpha \pi }}{2},i = 1,2,3,4,5,\alpha \in \left( {0,1} \right]$, $D^0$ is locally asymptotically stable. $\square$

#### Remarks 3.2

The value of $R_0$ is a critical determinant of RSV transmission dynamics. A value below 1 suggests limited transmission, while a value above 1 indicates the potential for significant and sustained spread. This insight is essential for understanding the epidemiology of RSV and for designing effective control and prevention strategies.

## Qualitative analysis of model

Here, we demonstrate that the system has a unique solution. System (3) is first written as follows:

10 $$\begin{aligned} \left\{ \begin{array}{l}^CD_t^\alpha S(t) = {P_1}(t, \, S(t)),\\ ^CD_t^\alpha E(t) = {P_2}(t, \, E(t)),\\ ^CD_t^\alpha {I_r}(t) = {P_3}(t, \, {I_r}),\\ ^CD_t^\alpha {I_s}(t) = {P_4}(t, \, {I_s}),\\ ^CD_t^\alpha R(t) = {P_5}(t, \, R).\end{array} \right. \end{aligned}$$

The aforementioned equations can be solved by applying integral form to both sides.

11 $$\begin{aligned} \left\{ \begin{array}{l}S(t) - S(0) = \frac{1}{{\Gamma (\alpha )}}\int \limits _0^t {{P_1}(t, \, S(t)){{(t - \sigma )}^{\alpha - 1}}d\sigma ,} \\ E(t) - E(0) = \frac{1}{{\Gamma (\alpha )}}\int \limits _0^t {{P_2}(t,\, E(t)){{(t - \sigma )}^{\alpha - 1}}d\sigma ,} \\ {I_r}(t) - {I_r}(0) = \frac{1}{{\Gamma (\alpha )}}\int \limits _0^t {{P_3}(t,\, {I_r}(t)){{(t - \sigma )}^{\alpha - 1}}d\sigma ,} \\ {I_s}(t) - {I_s}(0) = \frac{1}{{\Gamma (\alpha )}}\int \limits _0^t {{P_4}(t, \, {I_s}(t)){{(t - \sigma )}^{\alpha - 1}}d\sigma ,} \\ R(t) - R(0) = \frac{1}{{\Gamma (\alpha )}}\int \limits _0^t {{P_5}(t, \, R(t)){{(t - \sigma )}^{\alpha - 1}}d\sigma .} \end{array} \right. \end{aligned}$$

We demonstrate that the Lipschitz condition and contraction are satisfied by the kernels $P_i = 1, 2, 3, 4, 5$.

### Theorem 4.1

*If the following inequality holds, then the kernel* $P_1$ *fulfills both the Lipschitz condition and contraction*:

$$\begin{aligned} 0 \le \beta {k_1} + \beta {k_2} + \mu < 1. \end{aligned}$$

### *Proof*

We have

$$\begin{aligned} {}\left\| {{P_1}(t,\,S) - {P_1}(t, \,{S_1})} \right\|&= \left\| { - \left( {\beta {I_r}(t) + \beta {I_s}(t)} \right) \left( {S(t) - {S_1}(t)} \right) - \mu \left( {S(t) - {S_1}(t)} \right) } \right\| \\&\le \left\| {\beta {I_r}(t) + \beta {I_s}(t)} \right\| \left\| {S(t) - {S_1}(t)} \right\| + \mu \left\| {S(t) - {S_1}(t)} \right\| \\&\le \left( {\beta \left\| {{I_r}} \right\| + \beta \left\| {{I_s}} \right\| + \mu } \right) \left\| {S(t) - {S_1}(t)} \right\| \\ {}&\le \left( {\beta {k_1} + \beta {k_2} + \mu } \right) \left\| {S(t) - {S_1}(t)} \right\| . \end{aligned}$$

Assume that ${m_1} = \beta {k_1} + \beta {k_2} + \mu ,$ where $\left\| {{I_r}} \right\| \le {k_1},$ $\left\| {{I_s}} \right\| \le {k_2}$ are bounded functions.

Therefore

12 $$\begin{aligned} \left\| {{P_1}(t,\,S) - {P_1}(t,\,{S_1})} \right\| \le {m_1}\left\| {S(t) - {S_1}(t)} \right\| . \end{aligned}$$

As a result, $P_1$ meets the Lipschitz condition, and $P_1$ is a contraction if $0 \le \beta {k_1} + \beta {k_2 } + \mu < 1$. Similarly, we may demonstrate that $P_i, i = 2, 3, 4, 5$ fulfil the Lipschitz condition and $P_i$ are contractions for $i = 2, 3, 4, 5,$ if $0 \le m_i < 1,$ where ${m_2} = \left( {\frac{1}{\eta }} \right) p + \left( {\frac{1}{\eta }} \right) \left( {1 - p} \right) + \mu ,$ ${m_3} = {r_1} + \mu ,$ ${m_4} = {r_2} + \mu ,$ ${m_5} = \mu$ are bounded functions. Take into account the following recursive forms of system (11):

$$\begin{aligned} \begin{array}{l}{\varphi _{1n}}(t) = {S_n}(t) - {S_{n - 1}}(t) = \frac{1}{{\Gamma (\alpha )}}\int \limits _0^t {\left( {{P_1}(\sigma ,\,{S_{n - 1}}) - {P_1}(\sigma ,\,{S_{n - 2}})} \right) } {(t - \sigma )^{\alpha - 1}}d\sigma ,\\ {\varphi _{2n}}(t) = {E_n}(t) - {E_{n - 1}}(t) = \frac{1}{{\Gamma (\alpha )}}\int \limits _0^t {\left( {{P_2}(\sigma ,\,{E_{n - 1}}) - {P_2}(\sigma ,\,{E_{n - 2}})} \right) } {(t - \sigma )^{\alpha - 1}}d\sigma ,\\ {\varphi _{3n}}(t) = {I_{r_{n}}}(t) - {I_{r_{n-1}}}(t) = \frac{1}{{\Gamma (\alpha )}}\int \limits _0^t {\left( {{P_3}(\sigma ,\,{I_{r_{n-1}}} - {P_3}(\sigma ,\,{I_{r_{n-2}}})} \right) } {(t - \sigma )^{\alpha - 1}}d\sigma ,\\ {\varphi _{4n}}(t) = {I_{{s_n}}}(t) - {I_{s_{n - 1}}}(t) = \frac{1}{{\Gamma (\alpha )}}\int \limits _0^t {\left( {{P_4}(\sigma ,\,{I_{s_{n - 1}}}) - {P_4}(\sigma ,\,{I_{s_{n - 2}}})} \right) } {(t - \sigma )^{\alpha - 1}}d\sigma ,\\ {\varphi _{5n}}(t) = {R_n}(t) - {R_{n - 1}}(t) = \frac{1}{{\Gamma (\alpha )}}\int \limits _0^t {\left( {{P_5}(\sigma ,\,{R_{n - 1}}) - {P_5}(\sigma ,\,{R_{n - 2}})} \right) } {(t - \sigma )^{\alpha - 1}}d\sigma ,\end{array} \end{aligned}$$

with the initial circumstances ${S_0}(t) = S(0),$ ${E_0}(t) = E(0),$ ${I_{r_0}}(t) = {I_r}(0),$ ${I_{s_0}}(t) = {I_s}(0),$ ${R_0}(t) = R(0).$ Take the norm of the first equation of the above system

$$\begin{aligned} {}\left\| {{\varphi _{1n}}(t)} \right\|&= \left\| {{S_n}(t) - {S_{n - 1}}(t)} \right\| \\ {}&= \left\| {\frac{1}{{\Gamma (\alpha )}}\int \limits _0^t {\left( {{P_1}(\sigma ,\,{S_{n - 1}}) - {P_1}(\sigma ,\,{S_{n - 2}})} \right) } {{(t - \sigma )}^{\alpha - 1}}d\sigma } \right\| \\ {}&\le \frac{1}{{\Gamma (\alpha )}}\int \limits _0^t {\left\| {\left( {{P_1}(\sigma ,\,{S_{n - 1}}) - {P_1}(\sigma ,\,{S_{n - 2}})} \right) {{(t - \sigma )}^{\alpha - 1}}} \right\| } d\sigma , \end{aligned}$$

Lipschitz condition (12) gives us

13 $$\begin{aligned} \left\| {{\varphi _{1n}}(t)} \right\| \le \frac{1}{{\Gamma (\alpha )}}{m_1}\int \limits _0^t {\left\| {{\varphi _{1(n - 1)}}(t)} \right\| } d\sigma . \end{aligned}$$

Similar to this, we get

14 $$\begin{aligned} \left\| {{\varphi _{2n}}(t)} \right\|\le & {} \frac{1}{{\Gamma (\alpha )}}{m_2}\int \limits _0^t {\left\| {{\varphi _{2(n - 1)}}(t)} \right\| } d\sigma ,\nonumber \\ \left\| {{\varphi _{3n}}(t)} \right\|\le & {} \frac{1}{{\Gamma (\alpha )}}{m_3}\int \limits _0^t {\left\| {{\varphi _{3(n - 1)}}(t)} \right\| } d\sigma ,\nonumber \\ \left\| {{\varphi _{4n}}(t)} \right\|\le & {} \frac{1}{{\Gamma (\alpha )}}{m_4}\int \limits _0^t {\left\| {{\varphi _{4(n - 1)}}(t)} \right\| } d\sigma ,\nonumber \\ \left\| {{\varphi _{5n}}(t)} \right\|\le & {} \frac{1}{{\Gamma (\alpha )}}{m_5}\int \limits _0^t {\left\| {{\varphi _{5(n - 1)}}(t)} \right\| } d\sigma . \end{aligned}$$

Hence, we can state that

$$\begin{aligned} {S_n}(t) = \sum \limits _{j = 1}^n {{\varphi _{1j}}(t),}\, \,{E_n}(t) = \sum \limits _{j = 1}^n {{\varphi _{2j}}(t),} \, \,{I_{r_n}}(t) = \sum \limits _{j = 1}^n {{\varphi _{3j}}(t),} \, \, {I_{s_n}}(t) = \sum \limits _{j = 1}^n {{\varphi _{4j}}(t),} \, \, {R_n}(t) = \sum \limits _{j = 1}^n {{\varphi _{5j}}(t).} \end{aligned}$$

We demonstrate that a solution exists in the following theorem. $\square$

### Theorem 4.2

*A system of solutions of the model* (3) *exists if there is* $\xi _1$ *such that*

$$\begin{aligned} \frac{1}{{\Gamma (\alpha )}}{\xi _1}{m_i} < 1. \end{aligned}$$

### *Proof*

From Eqs. (13) and (14), we have

$$\begin{aligned} \left\| {{\varphi _{1n}}(t)} \right\|\le & {} \left\| {{S_n}(0)} \right\| {{\left[ {\frac{1}{{\Gamma (\alpha )}}\xi {m_1}} \right] }^n},\\ \left\| {{\varphi _{2n}}(t)} \right\|\le & {} \left\| {{E_n}(0)} \right\| {{\left[ {\frac{1}{{\Gamma (\alpha )}}\xi {m_2}} \right] }^n},\\ \left\| {{\varphi _{3n}}(t)} \right\|\le & {} \left\| {{I_{r_{n}}}(0)} \right\| {{\left[ {\frac{1}{{\Gamma (\alpha )}}\xi {m_3}} \right] }^n},\\ \left\| {{\varphi _{4n}}(t)} \right\|\le & {} \left\| {{I_{s_{n}}}(0)} \right\| {{\left[ {\frac{1}{{\Gamma (\alpha )}}\xi {m_4}} \right] }^n},\\ \left\| {{\varphi _{5n}}(t)} \right\|\le & {} \left\| {{R_n}(0)} \right\| {{\left[ {\frac{1}{{\Gamma (\alpha )}}\xi {m_5}} \right] }^n}. \end{aligned}$$

This means that the system is continuous and has a solution. Currently, we demonstrate how the aforementioned functions create a model solution (11). Considering that

$$\begin{aligned} \begin{array}{l}S(t) - S(0) = {S_n}(t) - {\Upsilon _{1n}}(t),\\ E(t) - E(0) = {E_n}(t) - {\Upsilon _{2n}}(t),\\ {I_r}(t) - {I_r}(0) = {I_{r_{n}}}(t) - {\Upsilon _{3n}}(t),\\ {I_s}(t) - {I_s}(0) = {I_{s_{n}}}(t) - {\Upsilon _{4n}}(t),\\ R(t) - R(0) = {R_n}(t) - {\Upsilon _{5n}}(t).\end{array} \end{aligned}$$

So

$$\begin{aligned} {}\left\| {{\Upsilon _{1n}}(t)} \right\|&= \left\| {\frac{1}{{\Gamma (\alpha )}}\int \limits _0^t {\left( {{P_1}(\sigma ,\, S) - {P_1}(\sigma , \,{S_{n - 1}})} \right) d\sigma } } \right\| \\&\le \frac{1}{{\Gamma (\alpha )}}\left\| {\int \limits _0^t {\left( {{P_1}(\sigma ,\, S) - {P_1}(\sigma , \,{S_{n - 1}})} \right) } } \right\| d\sigma \\ {}&\le \frac{1}{{\Gamma (\alpha )}}{m_1}\left\| {S - {S_{n - 1}}} \right\| \xi . \end{aligned}$$

The procedure is repeated to produce

$$\begin{aligned} \left\| {{\Upsilon _{1n}}(t)} \right\| \le {\left[ {\frac{1}{{\Gamma (\alpha )}}\xi } \right] ^{n + 1}}m_1^{n + 1}b. \end{aligned}$$

At $\xi _1,$ we get

$$\begin{aligned} \left\| {{\Upsilon _{1n}}(t)} \right\| \le {\left[ {\frac{1}{{\Gamma (\alpha )}}{\xi _1}} \right] ^{n + 1}}m_1^{n + 1}b. \end{aligned}$$

Limiting the current equation as *n* gets closer to $\infty$, we obtain $\left\| {{\Upsilon _{in}}(t)} \right\| \rightarrow 0,i = 2,3,4,5.$ Similarly, we may demonstrate that $\left\| {{\Upsilon _{in}}(t)} \right\| \rightarrow 0,i = 2,3,4,5.$ The proof is now complete. $\square$

We assume that the system has an alternative solution, such as $S_1(t),$ $E_1(t),$ $I_{r1}(t),$ $I_{s1}(t),$ and $R_1(t),$ then we have

$$\begin{aligned} S(t) - {S_1} = \frac{1}{{\Gamma (\alpha )}}\int \limits _0^t {\left( {{P_1}(\sigma ,\,S) - {P_1}(\sigma ,\,{S_{n - 1}})} \right) d\sigma }. \end{aligned}$$

We use the norm of the aforementioned equation

$$\begin{aligned} \left\| {S(t) - {S_1}} \right\| = \frac{1}{{\Gamma (\alpha )}}\int \limits _0^t {\left\| {\left( {{P_1}(\sigma ,\,S) - {P_1}(\sigma ,\,{S_{n - 1}})} \right) } \right\| d\sigma }. \end{aligned}$$

Lipschitz condition (12) implies that

$$\begin{aligned} \left\| {S(t) - {S_1}} \right\| \le \frac{1}{{\Gamma (\alpha )}}{m_1}\xi \left\| {S(t) - {S_1}} \right\| . \end{aligned}$$

Thus

15 $$\begin{aligned} \left\| {S(t) - {S_1}} \right\| \left( {1 - \frac{1}{{\Gamma (\alpha )}}{m_1}\xi } \right) \le 0. \end{aligned}$$

### Theorem 4.3

*If the following circumstance applies, the solution of model* (3) *is distinct*:

$$\begin{aligned} 1 - \frac{1}{{\Gamma (\alpha )}}{m_1}\xi > 0. \end{aligned}$$

### *Proof*

Assuming condition (15) is satisfied

$$\begin{aligned} \left\| {S(t) - {S_1}} \right\| \left( {1 - \frac{1}{{\Gamma (\alpha )}}{m_1}\xi } \right) \le 0. \end{aligned}$$

Then $\left\| {S(t) - {S_1}} \right\| = 0.$ So, we obtain $S(t) = S_1(t)$. Similar equality may be demonstrated for *E*,  $I_r,$ $I_s,$ *R*. $\square$

## Ulam–Hyers stability

In this section of the paper, we will examine the stability of the system (3) considering the perspective of UH. The analysis of approximate solution stability holds significant importance.

### Definition 5.1

The considered system is said to be UH stable if $\exists$ some constants ${\Upsilon _i} > 0,$ $i \in {N^5}$ and for each ${\Xi _i} > 0$, $i \in {N^5}$, for

$$\begin{aligned}{} & {} \left| {S(t) - \frac{1}{{\Gamma (\alpha )}}\int \limits _0^t {{{(t - \sigma )}^{1 - \alpha }}{P_1}(\sigma ,\, S(\sigma ))d\sigma } } \right| \le {\Upsilon _1},\\{} & {} \left| {E(t) - \frac{1}{{\Gamma (\alpha )}}\int \limits _0^t {{{(t - \sigma )}^{1 - \alpha }}{P_2}(\sigma , \,E(\sigma ))d\sigma } } \right| \le {\Upsilon _2},\\{} & {} \left| {{I_r}(t) - \frac{1}{{\Gamma (\alpha )}}\int \limits _0^t {{{(t - \sigma )}^{1 - \alpha }}{P_3}(\sigma , \,{I_r}(\sigma ))d\sigma } } \right| \le {\Upsilon _3},\\{} & {} \left| {{I_s}(t) - \frac{1}{{\Gamma (\alpha )}}\int \limits _0^t {{{(t - \sigma )}^{1 - \alpha }}{P_4}(\sigma , \,{I_s}(\sigma ))d\sigma } } \right| \le {\Upsilon _4},\\{} & {} \left| {R(t) - \frac{1}{{\Gamma (\alpha )}}\int \limits _0^t {{{(t - \sigma )}^{1 - \alpha }}{P_5}(\sigma ,\, R(\sigma ))d\sigma } } \right| \le {\Upsilon _5}, \end{aligned}$$

and there exist $\left\{ {{\tilde{S}},\,{\tilde{E}},\,{{{\tilde{I}}}_r},\,{{{\tilde{I}}}_s},\,{\tilde{R}}} \right\}$ satisfy the following

16 $$\begin{aligned} {\tilde{S}}(t)= & {} \frac{1}{{\Gamma (\alpha )}}\int \limits _0^t {{{(t - \sigma )}^{1 - \alpha }}{P_1}(\sigma ,\,{\tilde{S}}(\sigma ))d\sigma },\nonumber \\ {\tilde{E}}(t)= & {} \frac{1}{{\Gamma (\alpha )}}\int \limits _0^t {{{(t - \sigma )}^{1 - \alpha }}{P_2}(\sigma ,\,{\tilde{E}}(\sigma ))d\sigma },\nonumber \\ {{{\tilde{I}}}_r}(t)= & {} \frac{1}{{\Gamma (\alpha )}}\int \limits _0^t {{{(t - \sigma )}^{1 - \alpha }}{P_3}(\sigma ,\,{{{\tilde{I}}}_r}(\sigma ))d\sigma ,} \nonumber \\ {{{\tilde{I}}}_s}(t)= & {} \frac{1}{{\Gamma (\alpha )}}\int \limits _0^t {{{(t - \sigma )}^{1 - \alpha }}{P_4}(\sigma ,\,{{{\tilde{I}}}_s}(\sigma ))d\sigma ,} \nonumber \\ {\tilde{R}}(t)= & {} \frac{1}{{\Gamma (\alpha )}}\int \limits _0^t {{{(t - \sigma )}^{1 - \alpha }}{P_5}(\sigma ,\,{\tilde{R}}(\sigma ))d\sigma }, \end{aligned}$$

such that

$$\begin{aligned} \left| {S - {\tilde{S}}} \right| \le {\Upsilon _1}{\Xi _1},\,\left| {E - {\tilde{E}}} \right| \le {\Upsilon _2}{\Xi _2},\,\left| {{I_r} - {{{\tilde{I}}}_r}} \right| \le {\Upsilon _3}{\Xi _3},\,\left| {{I_s} - {{{\tilde{I}}}_s}} \right| \le {\Upsilon _4}{\Xi _4},\,\left| {R - {\tilde{R}}} \right| \le {\Upsilon _5}{\Xi _5}. \end{aligned}$$

### Assumption 5.1

Let us assume a Banach space on a real-valued function $\textrm{B}(u)$ and $u=[0, b]$ and $u = \textrm{B}(u) \times \textrm{B}(u) \times \textrm{B}(u) \times \textrm{B}(u) \times \textrm{B}(u)$ prescribe a norm ${\sup }_{t \in u}$ $\left\| {{\tilde{S}},\,{\tilde{E}},\,{{{\tilde{I}}}_r},\,{{{\tilde{I}}}_s},\,{\tilde{R}}} \right\| = {\sup }_{t \in u} \left| {{\tilde{S}}} \right| + {\sup }_{t \in u} \left| {{\tilde{E}}} \right| + {\sup }_{t \in u} \left| {{{{\tilde{I}}}_r}} \right| + {\sup }_{t \in u} \left| {{{{\tilde{I}}}_s}} \right| + {\sup }_{t \in u} \left| {{\tilde{R}}} \right| .$

### Theorem 5.1

*The considered system is UH stable with the above assumption*.

### *Proof*

The system has a unique solution, we have

17 $$\begin{aligned} \left\| {S - {\tilde{S}}} \right\|= & {} \frac{1}{{\Gamma (\alpha )}}\int \limits _0^t {{{(t - \sigma )}^{1 - \alpha }}\left\| {{P_1}(\sigma ,\,S(\sigma )) - {P_1}(\sigma ,\,{\tilde{S}}(\sigma ))} \right\| d\sigma } \nonumber \\{} & {} \le {\Delta _1}\left\| {S - {\tilde{S}}} \right\| . \end{aligned}$$

18 $$\begin{aligned} \left\| {E - {\tilde{E}}} \right\|= & {} \frac{1}{{\Gamma (\alpha )}}\int \limits _0^t {{{(t - \sigma )}^{1 - \alpha }}\left\| {{P_2}(\sigma ,\,E(\sigma )) - {P_2}(\sigma ,\,{\tilde{E}}(\sigma ))} \right\| d\sigma } \nonumber \\{} & {} \le {\Delta _2}\left\| {E - {\tilde{E}}} \right\| . \end{aligned}$$

19 $$\begin{aligned} \left\| {{I_r} - {{{\tilde{I}}}_r}} \right\|= & {} \frac{1}{{\Gamma (\alpha )}}\int \limits _0^t {{{(t - \sigma )}^{1 - \alpha }}\left\| {{P_3}(\sigma ,\,{I_r}(\sigma )) - {P_3}(\sigma ,\,{{{\tilde{I}}}_r}(\sigma ))} \right\| d\sigma }\nonumber \\{} & {} \le {\Delta _3}\left\| {{I_r} - {{{\tilde{I}}}_r}} \right\| . \end{aligned}$$

20 $$\begin{aligned} \left\| {{I_s} - {{{\tilde{I}}}_s}} \right\|= & {} \frac{1}{{\Gamma (\alpha )}}\int \limits _0^t {{{(t - \sigma )}^{1 - \alpha }}\left\| {{P_4}(\sigma ,\,{I_s}(\sigma )) - {P_4}(\sigma ,\,{{{\tilde{I}}}_s}(\sigma ))} \right\| d\sigma }\nonumber \\{} & {} \le {\Delta _4}\left\| {{I_s} - {{{\tilde{I}}}_s}} \right\| . \end{aligned}$$

21 $$\begin{aligned} \left\| {R - {\tilde{R}}} \right\|= & {} \frac{1}{{\Gamma (\alpha )}}\int \limits _0^t {{{(t - \sigma )}^{1 - \alpha }}\left\| {{P_5}(\sigma ,\,{\tilde{R}}(\sigma )) - {P_5}(\sigma ,\,{\tilde{R}}(\sigma ))} \right\| d\sigma } \nonumber \\{} & {} \le {\Delta _5}\left\| {R - {\tilde{R}}} \right\| . \end{aligned}$$

Using ${\Delta _i} = {\Upsilon _i}$ and $\frac{1}{{\Gamma (\alpha )}} = {\Xi _i}$, we have

$$\begin{aligned} \left\| {S - {\tilde{S}}} \right\| \le {\Upsilon _1}{\Xi _1}. \end{aligned}$$

Similarly, for the rest of the classes, we have the following

$$\begin{aligned}{} & {} \left\| {E - {\tilde{E}}} \right\| \le {\Upsilon _2}{\Xi _2},\\{} & {} \left\| {{I_r} - {{{\tilde{I}}}_r}} \right\| \le {\Upsilon _3}{\Xi _3},\\{} & {} \left\| {{I_s} - {{{\tilde{I}}}_s}} \right\| \le {\Upsilon _4}{\Xi _4},\\{} & {} \left\| {R - {\tilde{R}}} \right\| \le {\Upsilon _5}{\Xi _5}. \end{aligned}$$

Thus, we have completed the proof. $\square$

## Numerical scheme

The goal of this section is to create a numerical scheme for (3). To acquire computational results, the scheme has been used. Consider the following to illustrate this:

22 $$\begin{aligned} \left\{ \begin{array}{l}S(t) = S(0) + \frac{1}{{\Gamma (\alpha )}} \times \int \limits _0^t {{{(t - \sigma )}^{\alpha - 1}}} {A_1}(t,\,S,\,E,\,{I_r},\,{I_s},\,R)d\sigma ,\\ E(t) = E(0) + \frac{1}{{\Gamma (\alpha )}} \times \int \limits _0^t {{{(t - \sigma )}^{\alpha - 1}}} {A_2}(t,\,S,\,E,\,{I_r},\,{I_s},\,R)d\sigma ,\\ {I_r}(t) = {I_r}(0) + \frac{1}{{\Gamma (\alpha )}} \times \int \limits _0^t {{{(t - \sigma )}^{\alpha - 1}}} {A_3}(t,\,S,\,E,\,{I_r},\,{I_s},\,R)d\sigma ,\\ {I_s}(t) = {I_s}(0) + \frac{1}{{\Gamma (\alpha )}} \times \int \limits _0^t {{{(t - \sigma )}^{\alpha - 1}}} {A_4}(t,\,S,\,E,\,{I_r},\,{I_s},\,R)d\sigma ,\\ R(t) = R(0) + \frac{1}{{\Gamma (\alpha )}} \times \int \limits _0^t {{{(t - \sigma )}^{\alpha - 1}}} {A_5}(t,\,S,\,E,\,{I_r},\,{I_s},\,R)d\sigma .\end{array} \right. \end{aligned}$$

By employing Lagrange’s interpolation polynomial (LIP), we get

23 $$\begin{aligned} \begin{aligned} {}{S^{r + 1}}&= \frac{{{{\left( {\Delta t} \right) }^\alpha }}}{{\Gamma (\alpha + 1)}}\sum \limits _{\nu = 2}^r {{A_1}\left( {{t_{\nu - 2}},\,{S^{\nu - 2}},\,{E^{\nu - 2}},\,I_r^{\nu - 2},\,I_s^{\nu - 2},\,{R^{\nu - 2}}} \right) } \\&\quad \times \left[ {{{\left( {{\zeta _1}} \right) }^\alpha } - \left( {\zeta _2 } \right) } \right] \\&\quad + \frac{{{{\left( {\Delta t} \right) }^\alpha }}}{{\Gamma (\alpha + 2)}}\sum \limits _{\nu = 2}^r {\left[ \begin{array}{l}{A_1}\left( {{t_{\nu - 1}},\,{S^{\nu - 1}},\,{E^{\nu - 1}},\,I_r^{\nu - 1},\,I_s^{\nu - 1},\,{R^{\nu - 1}}} \right) \\ - {A_1}\left( {{t_{\nu - 2}},\,{S^{\nu - 2}},\,{E^{\nu - 2}},\,I_r^{\nu - 2},\,I_s^{\nu - 2},\,{R^{\nu - 2}}} \right) \end{array} \right] } \\&\quad \times \left[ {{{\left( {\zeta _1} \right) }^\alpha }\left( {\zeta _3 } \right) - {{\left( {\zeta _2 } \right) }^\alpha }\left( {\zeta _4 } \right) } \right] ,\end{aligned} \end{aligned}$$

24 $$\begin{aligned} \begin{aligned}{}{E^{r + 1}}&= \frac{{{{\left( {\Delta t} \right) }^\alpha }}}{{\Gamma (\alpha + 1)}}\sum \limits _{\nu = 2}^r {{A_2}\left( {{t_{\nu - 2}},\,{S^{\nu - 2}},\,{E^{\nu - 2}},\,I_r^{\nu - 2},\,I_s^{\nu - 2},\,{R^{\nu - 2}}} \right) } \\&\times \left[ {{{\left( {\zeta _1} \right) }^\alpha } - \left( {\zeta _2 } \right) } \right] \\&\quad + \frac{{{{\left( {\Delta t} \right) }^\alpha }}}{{\Gamma (\alpha + 2)}}\sum \limits _{\nu = 2}^r {\left[ \begin{array}{l}{A_2}\left( {{t_{\nu - 1}},\,{S^{\nu - 1}},\,{E^{\nu - 1}},\,I_r^{\nu - 1},\,I_s^{\nu - 1},\,{R^{\nu - 1}}} \right) \\ - {A_2}\left( {{t_{\nu - 2}},\,{S^{\nu - 2}},\,{E^{\nu - 2}},\,I_r^{\nu - 2},\,I_s^{\nu - 2},\,{R^{\nu - 2}}} \right) \end{array} \right] } \\&\quad \times \left[ {{{\left( {\zeta _1} \right) }^\alpha }\left( {\zeta _3 } \right) - {{\left( {\zeta _2 } \right) }^\alpha }\left( {\zeta _4} \right) } \right] ,\end{aligned} \end{aligned}$$

25 $$\begin{aligned} \begin{aligned}{}I_r^{r + 1}&= \frac{{{{\left( {\Delta t} \right) }^\alpha }}}{{\Gamma (\alpha + 1)}}\sum \limits _{\nu = 2}^r {{A_3}\left( {{t_{\nu - 2}},\,{S^{\nu - 2}},\,{E^{\nu - 2}},\,I_r^{\nu - 2},\,I_s^{\nu - 2},\,{R^{\nu - 2}}} \right) } \\&\times \left[ {{{\left( {\zeta _1} \right) }^\alpha } - \left( {\zeta _2 } \right) } \right] \\ {}&\quad + \frac{{{{\left( {\Delta t} \right) }^\alpha }}}{{\Gamma (\alpha + 2)}}\sum \limits _{\nu = 2}^r {\left[ \begin{array}{l}{A_3}\left( {{t_{\nu - 1}},\,{S^{\nu - 1}},\,{E^{\nu - 1}},\,I_r^{\nu - 1},\,I_s^{\nu - 1},\,{R^{\nu - 1}}} \right) \\ - {A_3}\left( {{t_{\nu - 2}},\,{S^{\nu - 2}},\,{E^{\nu - 2}},\,I_r^{\nu - 2},\,I_s^{\nu - 2},\,{R^{\nu - 2}}} \right) \end{array} \right] } \\ {}&\quad \times \left[ {{{\left( {\zeta _1} \right) }^\alpha }\left( {\zeta _3} \right) - {{\left( {\zeta _2} \right) }^\alpha }\left( {\zeta _4} \right) } \right] ,\end{aligned} \end{aligned}$$

26 $$\begin{aligned} \begin{aligned}{}I_s^{r + 1}&= \frac{{{{\left( {\Delta t} \right) }^\alpha }}}{{\Gamma (\alpha + 1)}}\sum \limits _{\nu = 2}^r {{A_4}\left( {{t_{\nu - 2}},\,{S^{\nu - 2}},\,{E^{\nu - 2}},\,I_r^{\nu - 2},\,I_s^{\nu - 2},\,{R^{\nu - 2}}} \right) } \\&\quad \times \left[ {{{\left( {\zeta _1} \right) }^\alpha } - \left( {\zeta _2} \right) } \right] \\&\quad + \frac{{{{\left( {\Delta t} \right) }^\alpha }}}{{\Gamma (\alpha + 2)}}\sum \limits _{\nu = 2}^r {\left[ \begin{array}{l}{A_4}\left( {{t_{\nu - 1}},\,{S^{\nu - 1}},\,{E^{\nu - 1}},\,I_r^{\nu - 1},\,I_s^{\nu - 1},\,{R^{\nu - 1}}} \right) \\ - {A_4}\left( {{t_{\nu - 2}},\,{S^{\nu - 2}},\,{E^{\nu - 2}},\,I_r^{\nu - 2},\,I_s^{\nu - 2},\,{R^{\nu - 2}}} \right) \end{array} \right] } \\&\quad \times \left[ {{{\left( {\zeta _1} \right) }^\alpha }\left( {\zeta _3} \right) - {{\left( {\zeta _1} \right) }^\alpha }\left( {\zeta _4} \right) } \right] ,\end{aligned} \end{aligned}$$

27 $$\begin{aligned} \begin{aligned}{}{R^{r + 1}}&= \frac{{{{\left( {\Delta t} \right) }^\alpha }}}{{\Gamma (\alpha + 1)}}\sum \limits _{\nu = 2}^r {{A_5}\left( {{t_{\nu - 2}},{S^{\nu - 2}},{E^{\nu - 2}},\,I_r^{\nu - 2},\,I_s^{\nu - 2},\,{R^{\nu - 2}}} \right) } \\&\quad \times \left[ {{{\left( {\zeta _1} \right) }^\alpha } - \left( {\zeta _2} \right) } \right] \\&\quad + \frac{{{{\left( {\Delta t} \right) }^\alpha }}}{{\Gamma (\alpha + 2)}}\sum \limits _{\nu = 2}^r {\left[ \begin{array}{l}{A_5}\left( {{t_{\nu - 1}},\,{S^{\nu - 1}},\,{E^{\nu - 1}},\,I_r^{\nu - 1},\,I_s^{\nu - 1},\,{R^{\nu - 1}}} \right) \\ - {A_5}\left( {{t_{\nu - 2}},\,{S^{\nu - 2}},\,{E^{\nu - 2}},\,I_r^{\nu - 2},\,I_s^{\nu - 2},\,{R^{\nu - 2}}} \right) \end{array} \right] } \\&\quad \times \left[ {{{\left( {\zeta _1} \right) }^\alpha }\left( {\zeta _3 } \right) - {{\left( {\zeta _1} \right) }^\alpha }\left( {\zeta _4 } \right) } \right] ,\end{aligned} \end{aligned}$$

where

${\zeta _1} = r - \nu + 1,$    ${\zeta _2} = r - \nu ,$  ${\zeta _3} = r - \nu + 3 + 2\alpha ,$   and ${\zeta _4} = r - \nu + 3 + 3\alpha .$ We included data from systematic literature reviews^13^. The following algorithm also presents the previously discussed methodology. It also mentions the output variables, initial conditions, set of parameters, and resulting equations.
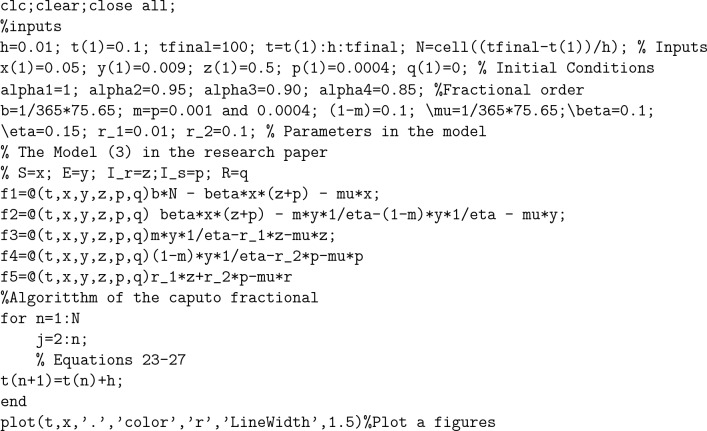


## Simulation and discussion

Here, we show how the transmission of RSV is impacted by fractional order. For a better understanding of the RSV infection phenomenon, we conducted several simulations. For numerical simulation, Table 1 contains the initial values of the compartments and the values of the model parameters.

**Table 1:** Parameters values and initial values of the compartments.

Parameter	Values	Parameter	Values	Source
*b*	$\frac{1}{{365 \times 75.65}}$	p	0.001 and 0.0004	^13^
$\mu$	$\frac{1}{{365 \times 75.65}}$	*S*(0)	0.05	^13^
${\beta }$	0.1	*E*(0)	0.009	^13^
${\eta }$	0.15	$I_r (0)$	0.5	^13^
$r_1$	0.01	$I_s$	0.0004	^13^
$r_2$	0.1	*R*	0	^13^

**Figure 1:**
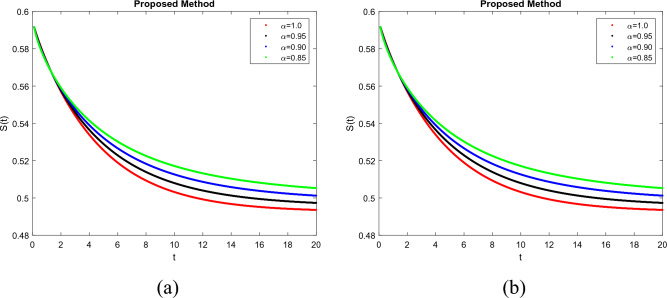
Simulation of S(t). (**a**) $p= 0.001$, (**b**) $p=0.0004$ under the Caputo model.

**Figure 2:**
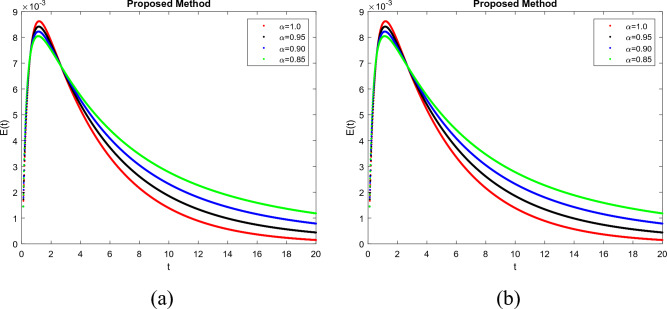
Simulation of E(t). (**a**) $p= 0.001$, (**b**) $p=0.0004$ under the Caputo model.

**Figure 3:**
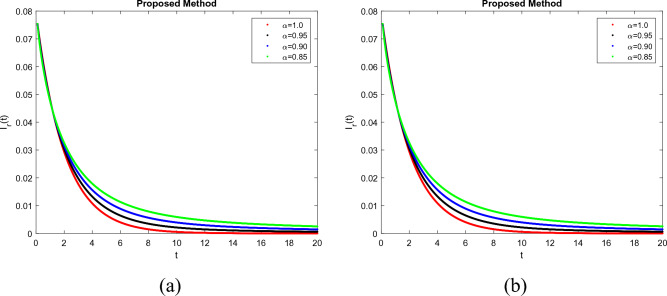
Simulation of $I_r(t)$. (**a**) $p= 0.001$, (**b**) $p=0.0004$ under the Caputo model.

**Figure 4:**
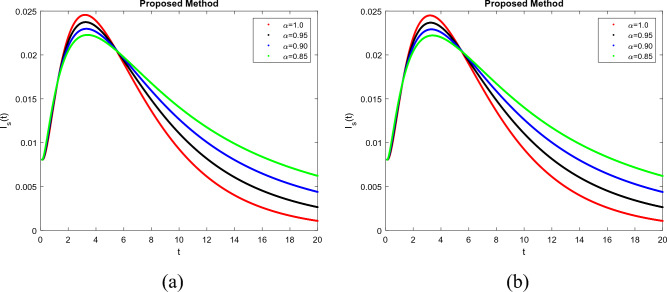
Simulation of $I_s(t)$. (**a**) $p= 0.001$, (**b**) $p=0.0004$ under the Caputo model.

**Figure 5:**
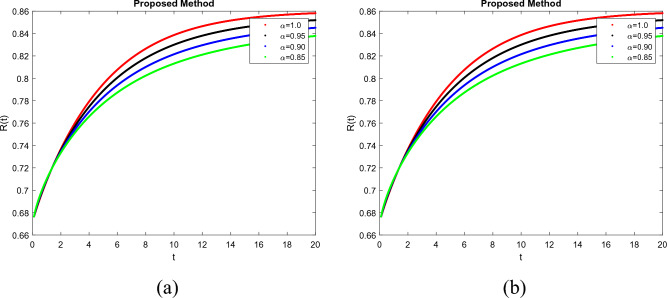
Simulation of R(t). (**a**) $p= 0.001$, (**b**) $p=0.0004$ under the Caputo model.

**Figure 6:**
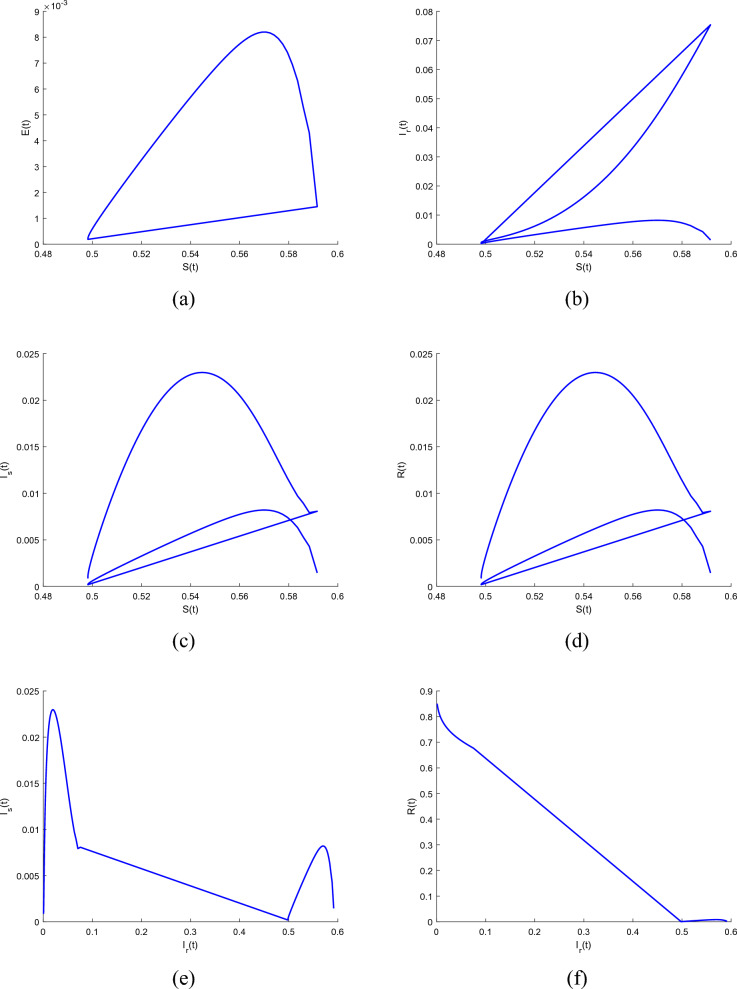
Simulation of chaotic behavior of compartments. (**a**) Behavior of *S*(*t*) and *E*(*t*). (**b**) Behavior of *S*(*t*) and $I_r(t)$. (**c**) Behavior of *S*(*t*) and $I_s(t)$. (**d**) Behavior of *S*(*t*) and *R*(*t*). (**e**) Behavior of $I_r(t)$ and $I_s(t)$. (**f**) Behavior of $I_r(t)$ and *R*(*t*).

To demonstrate how memory affects the dynamics of RSV, we consider various values of the memory index $(\alpha = 0.85, 0.90, 0.95, 1.00)$ in Figs. 1, 2, 3, 4 and 5. Figure 1 indicates that the susceptible population *S*(*t*) grows uniformly whenever the non-integer order $\alpha$ decreases. This phenomenon reflects how variations in memory index $\alpha$ influence immunity duration. A lower $\alpha$ value indicates stronger memory effects, causing individuals to retain immunity for an extended time after infection or vaccination. Figure 3 shows that, there is a sharp leap in the population of super spreading infected people in the early days when fractional order $\alpha$ decreases. This finding underscores the role of highly infectious individuals in initiating and driving early outbreaks. A decrease in memory index $\alpha$ could intensify the impact of memory-driven interactions, making highly infectious individuals more potent transmitters. This insight is crucial for anticipating and managing the initial surge of infection. Furthermore, as can be seen from Fig. 5 the recovered population *R*(*t*) decreases by decreasing memory index $\alpha$. A lower memory index $\alpha$ value implies a shorter duration of immunity post-recovery. This aligns with the idea that reduced $\alpha$ values emphasize stronger memory effects, resulting in faster waning of immunity. The observation emphasizes the potential for reinfections and the importance of immunity maintenance strategies. It is clear that the memory index has a significant impact on the dynamics of RSV and lowers the number of infected people. These observations reinforce the importance of memory effects, immunity, and initial transmission dynamics in the context of RSV infection. They offer insights that can influence public health strategies, vaccination programs, and the understanding of population-level immunity. The findings contribute to a more comprehensive understanding of the biological mechanisms underlying RSV behavior and its interactions with the human immune system, ultimately aiding in more effective disease management and control. Figs. 1, 2, 3, 4 and 5 depict the effects of input parameters p on the dynamics of RSV transmission, where the impact of infection progression rate has been established. Figure 6 shows the chaotic behavior of our system (3) with different settings for the memory index $\alpha$, the trajectories of the system converge to the equilibrium point. In Fig. 6a, we observe that the more individuals are susceptible the more transitioning to an exposed individuals whereas less the number of susceptible individuals the less the transitioning to an exposed individuals. In Fig. 6b, we observe that the more individuals are susceptible the more they get infected with RSV infection whereas less the number of susceptible individuals the less the number of individuals infected with RSV infection. Figure 6c describes that the number of recovered individuals decrease as the normal infected individuals increase. We noted that treatment effectiveness, immune response variations, and the overall progression of the infection collectively influence this chaotic transition. We observed that the memory index $\alpha$ can also be used as a chaotic control parameter. The chaotic behavior of the system is significantly relied upon in numerous scientific and engineering applications. It is generally known that there is a significant propensity to imagine and represent the behavior of chaotic systems. The proposed mathematical model is made feasible and scalable by the chaotic modeling, which can then be used to study novel chaos systems. We demonstrated how $\alpha$ might have made a significant contribution and could be used as a useful parameter for preventative measures.

## Conclusion

The most frequent cause of lower respiratory tract infections in newborns and children globally is respiratory syncytial virus (RSV). In this work, we have presented a fractional-order mathematical model for the respiratory syncytial virus (RSV) transmission in the presence of a super-spreader. Using the Caputo derivative, we provided the suggested model. The results demonstrate that the suggested model has bounded and positive solutions. The sensitivity analysis indicates that the value R0 correlates directly with the birth rate of susceptible individuals $(\mu )$, the virus transmission rate between humans $(\beta )$, and the likelihood of a new case being a normally infected human (*p*). These factors are adjustable through the efficient implementation of vaccination campaigns. Using the fixed-point theorem, we investigated the existence and uniqueness of the system’s solutions. Furthermore, we established UHS results for our system of viral infection RSV. We presented a two-step Lagrange polynomial numerical technique for addressing the Caputo fractional derivative to understand the dynamics of RSV. With varying input parameters, the chaotic graphs have been displayed. It has been demonstrated that the chaotic behavior of the proposed model is affected by fractional order $\alpha$. Adding the memory index $\alpha$ is expected to improve the system and could have been employed as a controlling parameter. Every fractional order model, in our opinion, has more information than the integer orders. For example, the integer order model will only have one solution, but the fractional order in an interval will have an infinite number of solutions. The beauty of these operators is that they can find new information for orders other than one while still approaching the integer order solution for orders that are close to one. In conclusion, the utilization of data-driven approaches in Respiratory Syncytial Virus (RSV) modeling proves pivotal in understanding the complexities of disease transmission and management.

In our future work, we plan to implement optimal control analysis on this model to reduce infection rates and increase the number of healthy individuals. The biological phenomenon can be described using real data by extending our model to a new generalized fractional derivative. Additionally, we will also apply the fractional operators to stochastic models.

## Data Availability

The datasets used and/or analysed during the current study available from the corresponding author on reasonable request.
