# Author Correction: Qualitative analysis and chaotic behavior of respiratory syncytial virus infection in human with fractional operator

**DOI:** 10.1038/s41598-024-54130-9

**Published:** 2024-02-13

**Authors:** Saba Jamil, Abdul Bariq, Muhammad Farman, Kottakkaran Sooppy Nisar, Ali Akgül, Muhammad Umer Saleem

**Affiliations:** 1 Institute of Mathematics, Khwaja Fareed University of Engineering and Information Technology, Rahim Yar Khan, Pakistan; 2 Department of Mathematics, Laghman University, Mehtarlam, Laghman 2701 Afghanistan; 3 Department of Mathematics, Faculty of Arts and Sciences, Near East University, Mersin, Turkey; 4 Department of Computer Science and Mathematics, Lebanese American University, Beirut, Lebanon; 5 Department of Mathematics, College of Science and Humanities in Alkharj, Prince Sattam Bin Abdulaziz University, Alkharj, Saudi Arabia; 6 Department of Mathematics, Art and Science Faculty, Siirt University, 56100 Siirt, Turkey; 7 Department of Mathematics, University of Education, Lahore, Pakistan

Correction to: *Scientific Reports* 10.1038/s41598-023-51121-0, published online 25 January 2024

The original version of this Article contained an error in the Acknowledgements section.

“Research Supporting Project number (RSP2023R167), King Saud University, Riyadh, Saudi Arabia.”

now reads:

“This study is supported via funding from Prince Sattam bin Abdulaziz University project number (PSAU/2023/R/1444).”

The original Article has been corrected.

